# Gene Therapy for Glioblastoma Multiforme

**DOI:** 10.3390/v17010118

**Published:** 2025-01-16

**Authors:** Smit Shah, Joshua Green, Shantelle A. Graff, Qi Li, John D. Heiss

**Affiliations:** 1 Neurology Department, School of Medicine, University of South Carolina, 15 Medical Park Rd., Columbia, SC 29203, USA; spshah031591@gmail.com; 2 Surgical Neurology Branch, NINDS, NIH 10 Center Drive, Bethesda, MD 20892, USA; joshuatessgreen@gmail.com (J.G.); shantelle.graff@nih.gov (S.A.G.); 3 Neuro-Oncology Branch, CCR, NCI, NIH, 37 Convent Drive, Bethesda, MD 20892, USA; qi.li2@nih.gov

**Keywords:** glioblastoma, gene, therapy, vector, survival

## Abstract

Glioblastoma multiforme (GBM) is a devastating, aggressive primary brain tumor with poor patient outcomes and a five-year survival of less than 10%. Significant limitations to effective GBM treatment include poor drug delivery across the blood–brain barrier, drug resistance, and complex genetic tumor alterations. Gene therapy uses a mechanism different from other GBM therapies to reduce tumor growth and enhance antitumor immunity. This review article will provide an update on various viral and nonviral vectors, their DNA and RNA cargoes, and how they genetically modify tumor cells and evoke therapeutic responses to GBM. The article explores the oncolytic and immunogenic effects of gene therapy agents. It reviews promising DNA transgenes, RNA inhibitors, and vectors for anti-GBM therapy. The possible benefits of combining gene therapy with standard GBM treatments will also be covered.

## 1. Introduction

### 1.1. Standard Treatment of Glioblastoma

Glioblastoma multiforme (GBM) has an abysmal prognosis. GBM’s median overall survival is about 20 months with the current standard of care, which is maximal safe surgery followed by concurrent radiotherapy and temozolomide chemotherapy [1]. Adding tumor treating fields (TTFs) may improve prognosis [2]. GBM recurs after current therapy because it is an aggressive neoplasm with complex genetic alterations. The World Health Organization (WHO) classifies primary GBM as a Grade 4 tumor with an unmutated IDH gene [3]. Further molecular testing has shown that EGFR amplification, TERT mutation, a gain of Chromosome 7, and a loss of Chromosome 10 also characterize GBM [4]. In patients with presumed GBM, a neurosurgeon performs a surgical procedure to obtain tissue for pathologic diagnosis and molecular characterization. Tumor resection via craniotomy is preferred to stereotactic needle biopsy because it supplies tumor cytoreduction. Maximal safe tumor resection removes as much tumor as possible without creating a disabling neurological deficit. Subsequent workup and management are recommended by a multidisciplinary neuro-oncology tumor board that considers the GBM’s molecular characteristics, genetic variants, epigenetic modifications like MGMT methylation, immunohistochemical markers, and the patient’s post-operative functional status assessed by the Karnofsky Performance Scale (KPS) [5]. In general, for GBM patients with a KPS less than 70 and methylated MGMT, TMZ with RT and the best supportive care are recommended. On the other hand, for GBM patients with a KPS of less than 70 and unmethylated MGMT, RT without TMZ and the best supportive care are offered because expression of the unmethylated MGMT gene by the tumor cell inactivates TMZ. Treatment of higher functioning GBM patients with KPS of 70 or more depends on their age. For 65- to 70-year-old patients, RT with concurrent and adjuvant TMZ is offered. For patients over 70, treatment consists of a short course of RT, with concurrent and adjuvant TMZ given only to patients with methylated MGMT. Treatment with alternating TTFs is an optional adjunctive treatment [6,7].

Factors limiting the effectiveness of GBM treatment include resistance to tumoricidal chemo- and radiotherapy, complex alterations in tumor genetics driving cellular proliferation, and inadequate drug delivery across the blood–brain barrier. Gene therapy has a different antitumor mechanism than chemo- and radiotherapy and may avoid resistance mechanisms that abrogate chemo- and radiotherapy effects. Gene therapy can reduce tumor growth and enhance antitumor immunity [8]. This review article will update the readers on viral and nonviral vectors, their DNA and RNA cargoes, and how they genetically modify tumor cells. We will discuss the oncolytic and immunogenic effects of promising vectors, DNA transgenes, and RNA inhibitors being tested for anti-GBM therapy. Finally, we describe beneficial ways gene therapy can be combined with standard GBM treatments [9].

### 1.2. Epidemiology, Radiology, Basic Molecular Profiling, and Pathology of GBM

GBMs are usually present in patients over 40 years of age, and their incidence peaks in patients in their late 60s and early 70s. GBM predominates in males, with a 3:2 male-to-female ratio. The GBM incidence in Europe and North America is 3–4 cases per 100,000 people [10]. Most GBMs present sporadically and without a known predisposing condition. Some GBM patients have a prior history of radiation exposure and presumed radiation-induced glioma formation [11]. Patients with GBM usually present with a focal neurological deficit, symptoms of increased intracranial pressure, or seizures. Infrequently, in less than 2% of cases, patients present with acute, stroke-like symptoms from intra-tumoral hemorrhage.

GBMs can arise anywhere within the brain parenchyma, including the cerebrum, cerebellum, and brainstem. The cerebrum is the most frequent location, and GBM is often found in the subcortical white matter, deep gray matter, temporal lobes, and crossing white matter tracts [12]. Macroscopically, GBMs diffusely infiltrate, have localized necrotic areas, and are poorly marginated. GBMs have a firm texture in brain areas where they infiltrate and internal soft, gelatinous regions where they are necrotic. GBMs have an off-white background color and yellow-to-brown discoloration in pockets of necrosis and cystic hemorrhage [10].

Histologically, glioblastoma multiforme consists of pleomorphic astrocytes with marked atypia and frequent mitoses, microvascular proliferation, and pseudopalisading necrosis. Angiogenesis drives the proliferation of permeable microvessels that allow intravascular contrast agents to cross the blood–brain tumor barrier, seen as postcontrast tumor enhancement on CT and MRI scans [12,13,14]. GBM has enhancement and endovascular proliferation that is rarely seen in low-grade glioma. Low-grade gliomas are hypercellular and sometimes surrounded by peritumoral edema. Giant-cell glioblastoma, gliosarcoma, and epithelioid glioblastoma are GBM variants with specific imaging characteristics and biological behaviors. GBM immunohistologic markers include EGFR, S100, Nestin, and p53 mutation. Negative GBM markers include H3K27 M, which is positive in diffuse midline glioma, and IDH-1/R132H mutation, which is positive in low-grade glioma [3].

GBM appears on T1-MRI as a hyper- to isointense mass with a central heterogeneous signal from necrosis and microhemorrhage [15]. About 85% of GBMs have irregularly distributed low-intensity signals from blood products [16]. Post-gadolinium enhancement usually has irregular boundaries and occurs at the tumor periphery [17]. On T2 FLAIR MRI, GBMs are hyperintense and surrounded by vasogenic edema. Flow voids are occasionally seen. GRE and SWI sequences detect calcium and iron from intratumoral hemorrhage within the tumor that distorts the local magnetic field (susceptibility effect). DWI imaging sequences show elevated DWI signals. ADC sequences typically show intermediate diffusion restriction that contrasts with lesser restriction in the vasogenic edema surrounding the tumor [18]. On MR perfusion imaging, GBM has high CBV (cerebral blood volume), consistent with its heightened vascularity. In contrast, the CBV of low-grade tumors is like that of surrounding brain tissue. On MR spectroscopy, GBM is characterized by an elevated choline peak from increased cell membrane turnover, an elevated lactate peak from anaerobic metabolism, and lipid peaks from tissue damage liberating membrane lipids. On MR spectroscopy, GBM also reduces the NAA (N-Acetyl Aspartate) peak by destroying neuronal tissue and the myo-inositol peak by opening the blood–tumor barrier and disrupting local osmotic regulation [19].

### 1.3. Rationale for Glioblastoma Gene Therapy

Gene therapy for GBM typically uses viral vectors designed to target and produce tumoricidal effects on GBM. Challenges to using gene therapy for GBM include restricted delivery of viral vectors to a small proportion of tumor cells through the blood–brain barrier, low tumor cell transgene transfection, and incomplete tumoricidal effect of the transgene in transfected cells. Foreign proteins of the viral vector and transgene can evoke an immune response in immune-competent patients. However, glioblastoma secretes factors suppressing the local and systemic immune responses to gene therapy vectors [20,21].

## 2. Materials and Methods

**Figure 1:**
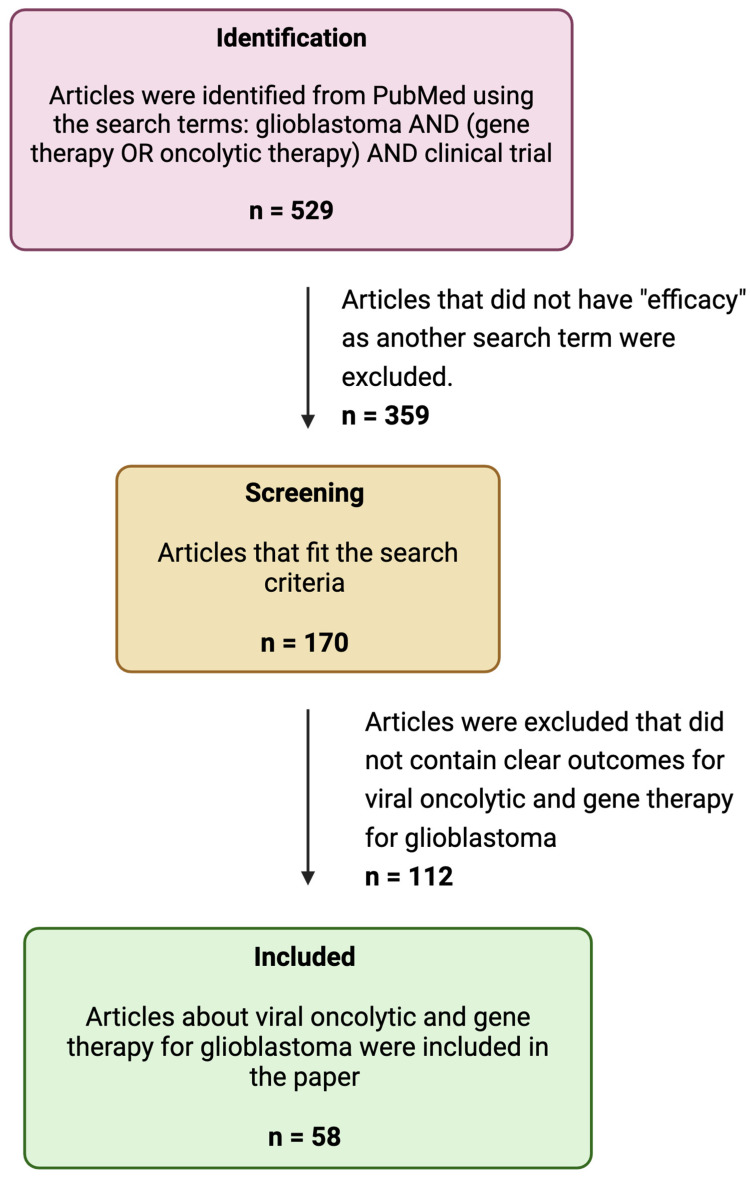
A flow diagram of the search strategy.

To write this review, we searched PubMed using the keywords “GBM”, “Gene Therapy”, “Oncolytic therapy”, and “Efficacy” for articles published on these subjects between 2000 and 2024 (Figure 1). From these articles, we selected articles related to viral oncolytic and gene therapy for GBM and reported them in tabular format (Table 1, Table 2 and Table 3).

Our review highlighted that gene therapy studies for GBM include (1) Phase I dose-escalation studies to assess the maximal acceptable dose of gene therapy vector and (2) Phases II and III studies that compare the efficacy of new gene therapy approaches with standard therapy in recurrent, resistant, and progressive GBM. Some of these studies combine existing therapies with gene therapy.

## 3. Gene Therapy for GBM Using Viral Vectors

### 3.1. Gene Therapy for GBM Using One Viral Vector

**Figure 2:**
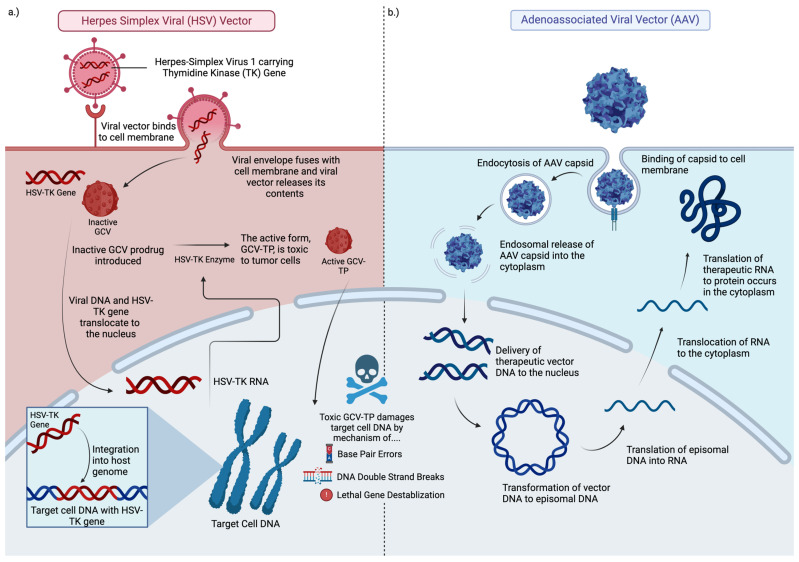
Graphic of viral vector gene therapy agents. Panel (**a**) depicts the mechanism of a modified Herpes Simplex Viral vector delivering the thymidine kinase transgene. The transgene integrates into the target cell host DNA before translating mRNA in the nucleus, which expresses the TK enzyme within the cytoplasm. Ganciclovir (GCV) is taken up by the GBM cell. The TK enzyme phosphorylates GCV in the cytoplasm to its toxic form, ganciclovir triphosphate (GCV-TP), which damages DNA resulting in the death of the GBM cell. Panel (**b**) depicts the mechanism of a therapeutic adeno-associated viral vector delivering a transgene that transcribes its episomal DNA in the nucleus to mRNA, which translates the therapeutic RNA in the cytoplasm to the anti-neoplastic protein of interest. This figure incorporates features of figures in articles by Mendell [45] and Nicholas [46].

The Herpes simplex virus type I thymidine kinase (HSV 1–TK) transgene was tested in early gene therapy trials for GBM. Thymidine kinase (TK) is an enzyme that phosphorylates the prodrug valacyclovir and converts it to a nucleotide analog capable of killing dividing GBM cells (Figure 2a).

Adenovirus-based gene therapy vectors are being tested in oncology (Figure 2b). Some examples include Onyx–015, deltoid 24-h GD, DN X–2440, AVV-CMV-HSV-tk, Ad-hCMV-Flt3L, Ad.hIFN-β, VB-111, and Ad-RTS-hIL12. Adenovirus-virus vectors for gene therapy are immunogenic and may elicit immune-related antitumor effects and benefits. A phase 1 trial using an adenoviral vector carrying the p53 gene (Ad-p53) showed successful gene introduction into tumor cells, minimal toxicity, and limited tissue penetration. Another study compared ganciclovir combined with HSV-tk gene delivery via retrovirus or adenovirus in malignant glioma patients, finding that adenovirus-treated patients had more stable tumors and nearly double the survival time compared to retrovirus-treated patients. Additional studies explored an adenoviral vector with the HSV-tk gene (AdV-tk) combined with valacyclovir. This treatment converts the prodrug into toxic compounds, killing tumor cells and activating immune cells. A Phase 1B study of recently diagnosed malignant glioma patients showed improved two and three-year survival rates when AdV-tk was used with valacyclovir, radiation, and TMZ. CD3+ T-cells, showing immune activation, were found in treated tumors. A phase 2 trial showed a significant increase in median survival time from 13.5 months with standard care to 17.1 months with the addition of AdV-tk and valacyclovir [22].

Toca 511 and TG6002 are recombinant vaccinia viral vectors that carry a yeast cytosine deaminase (CD) gene. Gene transfection leads to the expression of CD in the GBM cells. Following 5-fluorocytosine (5-FC) delivery to GBM cells, CD converts 5-FC to 5-fluorouracil (5-FU), which is toxic to rapidly dividing cells. Another virus, MV-CEA, is a recombinant Edmonston strain of measles virus that binds to cell surface CD 64, is internalized, and causes cell lysis. Pelareorep (Reolysin) is a human wild-type reovirus. This non-enveloped double-stranded RNA virus uses the Ras pathway to cause tumor cell lysis [23] (Table 1). 

### 3.2. Gene Therapy for GBM Using Two Viral Vectors

Adenoviral vectors can carry the HSV1-TK gene to kill tumor cells or the FMS-like tyrosine kinase 3 ligand (Flt3L) gene to stimulate an anti-GBM immune response. Dual-vector treatment expressing HSV1-TK (Ad-hCMV-TK) and Flt3L (Ad-hCMV-Flt3L) was tested in mouse and rat GBM models. These preclinical studies showed that this dual-vector gene therapy induced T-cell-mediated, anti-GBM toxicity in most tumors. The HSV1-TK adenoviral vector transcribed the TK transgene in GBM cells. The HSV-TK enzyme converts monophosphorylated to triphosphorylated ganciclovir in transfected GBM tumor cells, which acts on the transfected and bystander GBM cells, inhibiting their DNA synthesis and causing their apoptosis [24]. Glioma antigens released by dying tumor cells evoked a robust anti-GBM immune response by recruiting infiltrating dendritic cells into the tumor microenvironment. Ad-hCMV-Flt3L augments the immune response. The safety of this dual vector gene therapy was tested in humans in a Phase I clinical trial. In this trial, an adenovirus carrying the TK gene (AD-hCMV-TK) and another carrying Flt3L (AD-hCMV-Flt3L) were coinjected into newly diagnosed high-grade gliomas, including GBM, followed by two 14-day courses of oral ganciclovir. The combination of the two adenoviral vectors was well-tolerated. The maximum tolerated viral vector dosage was not reached. The investigators suggested further evaluating this approach in a Phase 1b or 2 clinical trial [9].

### 3.3. Alphaviruses in GBM Therapy

**Figure 3:**
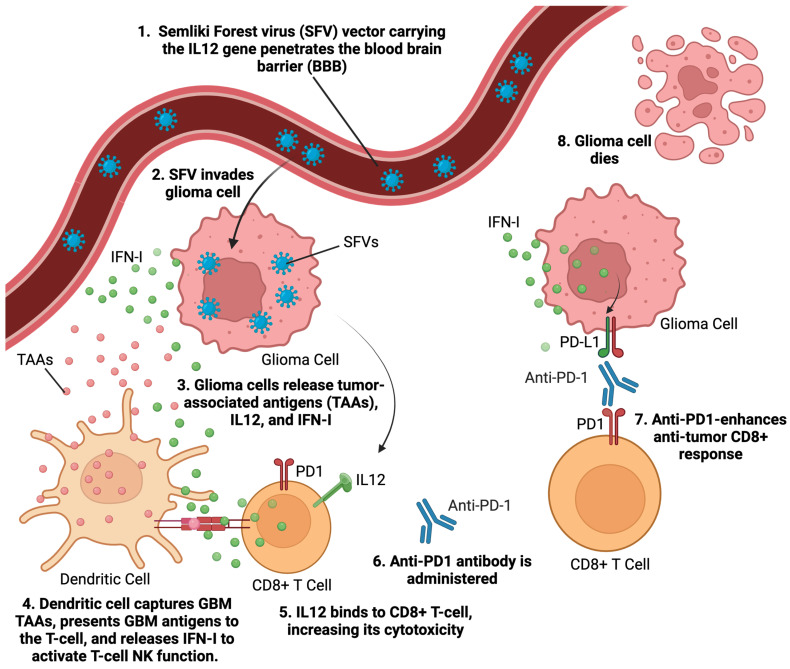
Graphic illustrating alphavirus mechanism of action. Semliki Forest virus (SFV), an alphavirus carrying the IL12 gene, effectively crosses the blood–brain barrier (BBB) to infect glioma cells. As SFV infects and induces apoptosis in glioma cells, the cells rupture, releasing tumor-associated antigens (TAAs) into the surrounding environment. These TAAs are captured by dendritic cells, which then present to CD8+ T-cells, priming T-cells to target and destroy glioma cells. Additionally, interferon type (IFN-I) is released from both the dying glioma cells and adjacent immune cells, further enhancing the immune response against the tumor. Anti-PD1 therapy enhances the immune response by blocking the PD1 receptor on T-cells, preventing exhaustion of the immune system. This inhibition amplifies the overall anti-tumor response that is triggered by SFV infection. This figure incorporates features of a graphical abstract by Martikainen et al. [30].

Alphaviruses are enveloped single-stranded RNA viruses [25]. They are self-replicating RNA viruses that amplify their RNA enormously, about 200,000-fold, in the cytoplasm of infected host cells [25]. Alphaviruses generate superior production of antigens or other proteins of interest compared to other viral vectors, which allows them to produce the same immunization effects as other viral vectors but at significantly reduced viral doses. Alphavirus vectors’ capability to produce desired effects at lower viral vector dosages potentially reduces their vector-related adverse effects. Semliki Forest virus (SFV) [26] is an alphavirus that has been used for GBM therapy. SFV particles expressing IL-12 (SFV-IL-12) demonstrated significant tumor reduction and survival benefits in GBM models [27] (Figure 3). The replication-proficient SFV(A774nsP) vector displayed potent oncolytic effects in GBM models, with long-term survival in treated animals [28]. Strategies like inserting neuron-sparing miRT124 sequences into SFV4 vectors have improved tumor targeting and survival in GBM models [29]. Additionally, SFV-AM6-124T can overcome hurdles of innate anti-viral signaling. Combination therapy with SFV-AM6-124T and anti-PD1 promotes the inflammatory response and improves the immune microenvironment in the GBM model [30]. Clinical studies on alphavirus-based treatment of GBM will begin after further preclinical developmental studies address safety concerns and optimize targeting strategies.

### 3.4. Genetically Modified Viruses for Oncolytic Therapy of GBM

Oncolytic viruses are being tested against GBM. These viruses are not gene therapy vectors but are viruses genetically modified to enhance their specificity, cytotoxicity, and immunogenicity to tumor tissue. A Phase I-II trial of viral oncolytic therapy used G47Δ, a triple-mutated, third-generation oncolytic herpes simplex virus type 1 (HSV-1). This virus was constructed by deleting HSV’s α47 gene and overlapping the US11 promoter from parental G207. It showed more tumor-specific replication capability, cytopathic effects, and a better safety profile than earlier HSV 1 oncolytic viruses. It was confirmed safe in a first-in-human (FIH) trial when administered intratumorally in two doses every 2 weeks to patients with recurrent GBM [15].

In a Phase 2 trial of oncolytic herpes virus G47Δ in patients with residual or recurrent GBM, the interim analysis of 13 patients revealed a 1-year survival rate of 92.3% (95% confidence interval [CI], 64.0–99.8) following G47∆ initiation, significantly higher than the preset control value of 15%. A total of 19 out of 28 patients meeting the inclusion criteria were enrolled, forming the complete analysis set (CAS). The primary endpoint was the 1-year survival rate, while secondary endpoints included overall survival (OS) and progression-free survival (PFS) after G47∆ initiation. For the CAS population, the 1-year survival rate was 84.2% (95% CI, 60.4–96.6). The median OS was 20.2 months (16.8–23.6 months) after G47∆ initiation, and the median PFS was 4.7 months (3.3–6.1 months) after G47Δ initiation. In terms of safety, a secondary endpoint, all 19 patients (100%) experienced G47∆-related adverse events such as fever (89.5%), vomiting (57.9%), nausea (52.6%), lymphocytopenia (47.4%), and leukopenia (31.6%). All patients recovered from these adverse events. The only serious adverse event was a grade 2 fever in one patient (5.3%), which led to a prolonged hospital stay. This trial highlights the efficacy and safety of G47Δ for treating residual or recurrent glioblastoma. The 1-year survival rate of 84.2% and median OS and PFS of 20.2 months and 4.7 months, respectively, of G47Δ compare favorably with other residual or recurrent GBM treatments [8]. Preclinical studies showed that G47∆ had immediate oncolytic effects from viral replication and later effects from evoking specific antitumor immunity [8].

HSV 1716 is another widely researched oncolytic, recombinant, replication-competent HSV 1 virus. Other HSV-based therapeutics include C134 oncolytic HSV-1, RL 1 gene, G207, M032–HSV 1, and rQNestin34.5v.2 [23]. Other viruses for viral oncolytic therapy include Newcastle disease virus, a single-stranded enveloped RNA virus that can induce apoptosis. ParvOryx, H-1PV, is an oncolytic wild-type parvovirus that highjacks host enzymes expressed during the S-phase of the cell cycle, making it selective for cancer cells and other rapidly dividing cells. PVSRIPO is a poliovirus type 1 (Sabin type) viral vector that binds to the cell surface receptor CD155, is internalized, and lyses tumor cells.

Viral gene therapy vectors and oncolytic viruses introduce foreign antigens that can activate the immune system [31]. Immune responses should be suppressed initially until after viral transfection in gene therapy or direct cytotoxic effects in viral oncolytic therapy. After the transgene or virus has a direct oncolytic effect on tumor cells, an unrestrained immune response to viral antigens or dying tumor cells can produce additional antitumoral effects.

## 4. Gene Therapy for GBM Using Nonviral Vectors

**Figure 4:**
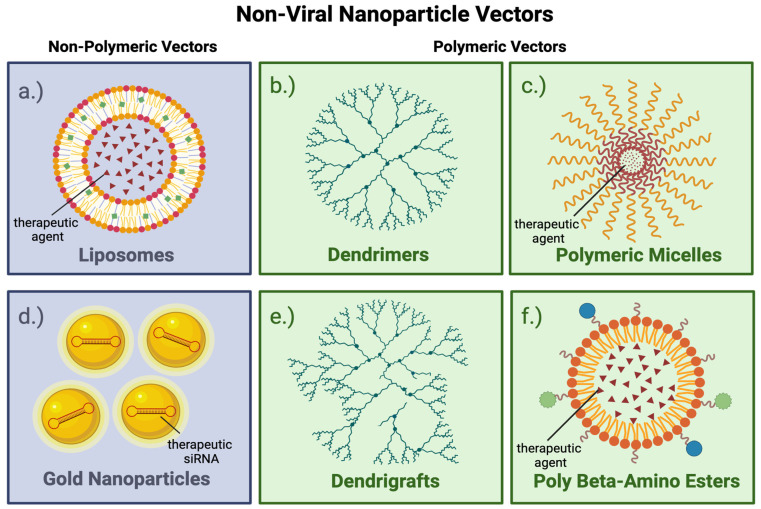
Graphic summary of nonviral nanoparticle vectors. Panels (**a**–**f**) illustrate different types of nonviral nanoparticle vectors. Each vector has an inner core surrounded by an outer shell. The core holds a therapeutic agent. The shell is an outer surface that can be modified to increase the nanoparticle’s ability to cross the blood–brain barrier to enter tumor cells. Nonviral vectors can be engineered by linking antibodies or other molecules to their surfaces to enhance their targeting specificity. All vectors can be modified on their surfaces by cell-targeting molecules, as shown in panel (**f**). The vectors bind to specific receptors on target cells, are internalized, and deliver the therapeutic agent.

Several nonviral approaches for delivering gene therapy to GBM are being explored as alternatives to viral vectors (Table 2). An ideal delivery system should protect the genetic therapy payload, such as mRNA, siRNA, or a DNA gene, from enzymatic degradation and excretion and promote its uptake into the cells and tissues of interest [32]. Polymeric and non-polymeric nonviral genetic vectors have undergone testing in preclinical and human studies. Polymeric molecules consist of monomeric subunits linked together in a network or chain. Dendrimers, dendrigrafts, polymeric micelles, and poly (β-amino esters) are polymers with structures to carry genetic cargoes and act as gene therapy vectors. Liposomes and gold NPs are non-polymeric genetic vectors (Figure 4). 

### 4.1. Gene Therapy for GBM Using Polymeric Vectors

Polymeric micelles are amphiphilic compounds, like liposomes, that form a shell around a core that carries gene therapy agents (Table 3). Cationic and hydrophobic polymer micelles can carry negatively charged nucleic acids and hydrophobic cancer drugs, respectively, in their cores [22]. Dendrimers are highly branched 3D polymers that carry genetic cargoes in their cores. Cationic dendrimers such as poly(amidoamine) (PAMAM) can form complexes with negatively charged nucleic acids. The cationic dendrimer surface helps penetrate cellular and endosomal membranes. Cationic dendrimers can cross the BBB and carry an array of gene therapy agents like antisense oligonucleotides, microRNAs, siRNAs, and DNA to glioma cells [22]. Dendrigrafts are dendrimers with molecules conjugated to their surfaces, targeting the cells of interest. Dendrigrafts, like dendrimers, can deliver genetic therapies. Dendrigraft poly-L-lysine (DGL) has external amino acid groups to conjugate the dendrigraft to transferrin or laminin. These molecules attach to their respective receptors, helping transport the dendrigraft’s genetic cargo across the BBB and target glioma cells. Initial studies involving dendrigrafts show they can deliver nucleic acids to glioma cells. Dendrigraft use is relatively new and evolving [22].

Polymeric nanoparticles are another type of gene therapy vector. Poly-β-amino esters are cationic polymers designed for gene delivery. Extensive polymer libraries can be synthesized using combinatorial chemistry to vary amine composition between PBAE polymers. High-throughput screening of hundreds of polymers can find which polymer is the best vector for gene delivery to tumor tissue. PBAE vectors in vitro can transfect up to 90% of primary GBM cells and silence up to 85% of genes with minimal cytotoxicity. Polymers are biodegradable and have low cytotoxicity [22].

Polyethylene glycol-polyethyleneimine (PEG-PEI) polymeric nanoparticles have undergone preclinical testing. Zhan and colleagues showed effective delivery of the TRAIL gene to intracranial GBM using RGD–PEG–PEI in mice. RGD is a crucial binding motif for the αvβ3 and other integrin receptors. The RGD ligand binds to integrin receptors and facilitates TRAIL gene transit across the blood–tumor barrier (BTB) and into the GBM. In this study, co-delivery of the TRAIL gene and Paclitaxel (PTX) significantly prolonged the survival of mice models of GBM [33].

Polyurethane (PU) is another polymeric vector that can be used for genetic therapy. In one study, PU-PEI complexed with miR145 delivered miR145 to over 90% of GBM CD133+ cells. The miR145 targeted the 3′UTRs of Oct4 and Sox2 and downregulated them [34]. Oct4 (Octamer-binding transcription factor 4) and SOX2 (Sex-determining region Y-box 2) are essential transcription factors for maintaining stem cell pluripotency and differentiation. In GBM, OCT4 and SOX2 induce an immune-suppressive state that allows tumor recurrence. PU-PEI complexed with miR145 inhibited GBM proliferation, altered cellular morphology, reduced invasiveness, and suppressed tumorigenesis [34,35].

Another nonviral vector is the multifunctional carrier (1-aminoethyl)iminobis[N-(oleicylcysteinylhistinyl-1-aminoethyl) propionamide) (EHCO). RGD/EHCO/siRNA, bombesin/EHCO/siRNA, and PEG-modified RGD/EHCO/siRNA nanoparticles were compared to free siRNA in mice tumor models. The RGD peptide (arginine, glycine, and aspartic acid) binds to cell surface integrin receptors [36]. Bombesin is a 14-amino acid polypeptide that binds to G-protein-coupled receptors, which are overexpressed in many human tumor types [37]. Cancer cells are hypoxic and induce hypoxia-inducible factor-1α (HIF-1α), vascular endothelial growth factor expression, angiogenesis, and tumor growth. The siRNA inhibited HIF-1α and tumor growth. The specific pH-sensitive amphiphilicity of the EHCO nanoparticles facilitated the endosomal escape of the siRNA cargo. Fourteen days after the first treatment, the average tumor sizes of mice treated with RGD-targeted nanoparticles, bombesin-targeted nanoparticles, pegylated nanoparticles, and free siRNA were 84.8%, 100.9%, 152.5%, and 170.2% of their initial sizes, respectively. The peptide-targeted siRNA/EHCO nanoparticles significantly suppressed tumor growth compared to free siRNA (*p* < 0.05) over the two-week treatment period [34,38] (Table 4).

### 4.2. Gene Therapy for GBM Using Non-Polymeric Vectors

NU-0129, a spherical gold nanoparticle with a nucleic acid core, carries small interfering RNAs (siRNAs) targeting the oncogenic Bcl2-like protein that supports tumor progression and apoptosis resistance [22]. SGT-53 is a liposomal vector that can cross the blood–brain barrier (BBB) to target GBM cells [22]. This liposomal vector holds wild-type p53 plasmid DNA in its core and displays transferrin on its surface. The surface transferrin molecule targets the transferrin receptor on the blood–brain barrier (BBB) and GBM cells. This vector carried the p53 gene, restored p53 function, and sensitized otherwise refractory tumor cells to an anti-programmed cell death protein (PD1) antibody [22]. Restoring p53 function also boosted antitumor immunity, reduced immune-related adverse events, and successfully augmented anti-PD1 therapy [39] (Table 5).

### 4.3. Gene Therapy for GBM Using Protein, Peptide, and Lipoprotein Vectors

Synthetic high-density lipoprotein and albumin-based nanoparticles (NPs) have also been studied [40,41]. Unmodified NP delivery systems have limited crossing of the blood–brain barrier (BBB), brain–tumor barrier (BTB), and vasculature. However, modifying the NP surface can improve drug delivery across the BBB and BTB to target tissues.

Albumin-based synthetic protein nanoparticles (SPNPs) can be engineered to reach intracranial GBMs after systemic delivery. Attaching the cell-penetrating peptide, RGD, to bind to integrin αVβ3, a protein involved in invasion and angiogenesis that is overexpressed in GBM, allows these SPNPs to cross the BBB and BTB and distribute throughout the tumor. Combining SPNP delivery with focused radiotherapy was highly tumoricidal, achieving long-term survival in 87.5% of treated mice [40]. Synthetic HDL (high-density lipoprotein) also resulted in a similar experimental outcome in mice studies with an 80% long-term survival [41]. Animal models in both studies remained tumor-free upon rechallenge with tumor cells in the contralateral hemisphere, confirming that immunologic memory had developed against GBM [40,41]. Albumin-coated peptide nanocomplexes have been developed to co-deliver temozolomide and the p53 gene to glioblastoma. In vitro experiments showed the nanocomplexes significantly reduced glioblastoma cell viability without toxicity to normal brain cells [42].

Stimuli-response peptide nanoparticles have also been developed that are loaded with siRNA cargoes which co-inhibit RELA/P65 and EGFR. These biodegradable nanoparticles have been tested in vitro and in vivo as GBM radiosensitizers. Their design incorporated αvβ3 integrin targeting to traverse the blood–brain barrier and pH-responsive endosomal escape to enhance drug delivery in tumor cells. After irradiation of U87MG-Luc glioblastoma cells in vitro, the GBM cells treated with these nanoparticles and radiation had a 37% apoptosis rate compared to 27% with radiation alone. In vivo, combining the nanoparticles with radiation produced a more robust anti-glioblastoma effect than nanoparticles or radiation alone [43] (Table 5).

### 4.4. Gene Therapy for GBM Using Magnetic Nanoparticles

In addition to targeted delivery, magnetic NPs allow non-invasive monitoring with MRI in real time of the location of NP composites carrying therapeutic elements. Veiseh and colleagues developed a nanoparticle delivery system with a super-paramagnetic iron oxide core covered by a positively charged copolymer of chitosan-grafted polyethylene glycol (PEG) and polyethyleneimine (PEI). Adding an siRNA effector molecule and a tumor-targeting peptide called chlorotoxin (CTX) to the nanoparticle makes it effective and specific in targeting tumors [44]. They experimented on a cell line, C6/GFP+, derived from mice glioma (C6) that was genetically modified to express green fluorescent protein (GFP). When C6/GFP+ cells were treated with NP-CTX, there was no significant change in GFP expression compared to untreated cells, meaning NP and CTX alone did not affect GFP. However, cells treated with NP-siRNA showed a 35% decrease in GFP expression. Importantly, cells treated with NP-siRNA-CTX had a substantial 62% reduction in GFP expression. The reduced GFP levels in C6/GFP+ cells showed that CTX receptor-mediated uptake of NP-siRNA-CTX increased the tumoricidal effects of the NP’s siRNA cargo [34,44].

**Table 1:** Overview of viral vectors and their characteristics for GBM gene therapy.

Viruses	Genome	Key Characteristics	Reference
Adenovirus	dsDNA	-Viral proteins facilitate endosomal escape and nuclear genome delivery. -Broad host range, infecting both dividing and non-dividing cells. -Short-term gene expression. -Strong immunogenicity.	[47]
Adeno associated viruses (AAVs) [48]	ssDNA	-Transduce cells via episomal transgene expression or random chromosomal integration. -Non-toxic, minimal inflammatory response.	Adeno associated viruses (AAVs) [48]
Retroviruses (classic) [49]	ssRNA	-RNA genome is retro-transcribed into linear double-stranded DNA and integrated into host chromatin. -Requires dividing cells for infection. -Enable long-term expression.	Retroviruses (classic) [49]
Lentiviruses [50]	ssRNA	-Subgroup of retroviruses that infect both dividing and non-dividing cells. -Utilizes active nuclear import, enabling host genome integration. -Stable and sustained transgene expression.	Lentiviruses [50]
Herpes simplex virus (HSV) [51]	dsDNA	-High vector capacity (~30 kb), allowing delivery of large genes or multiple transgenes. -Minimal integration into host DNA. -Establishes latent infection.	Herpes simplex virus (HSV) [51]
Alphavirus [52]	ssRNA	-SFV particles express IL-12. -Replication-proficient SFV(A774nsP) vector displayed potent oncolytic effects. -miRT124 sequence insertion into SFV4 vectors improves tumor targeting and survival. -SFV-AM6-124T overcomes innate anti-viral signaling. -Combined SFV-AM6-124T and anti-PD1 promotes inflammatory response and improves the immune microenvironment in the GBM model.	[25,26,27,28,29,30]

**Table 2:** Comparison of nonviral vectors for gene delivery in GBM gene therapy.

Nonviral Vector	Advantages	Limitations
Liposomes	-Biodegradable and non-cytotoxic. -Low immunogenicity. -Capable of encapsulating both hydrophilic and hydrophobic drugs.	-Short shelf life and susceptibility to degradation during storage. -Transient gene transfection, leading to temporary therapeutic effects. -Short half-life in systemic circulation, limiting in vivo applications.
Gold Nanoparticles	-Non-biodegradable but highly stable in biological environments. -Functionalize with biomolecules such as peptides, antibodies, and DNA for targeted delivery.	-Lack of biodegradability may lead to long-term accumulation and potential toxicity. -Requires careful design to minimize adverse side effects in vivo.
Dendrimers	-Highly branched structure allows for precise functionalization, high drug-loading capacity, and targeting. -Non-immunogenic.	-Limited drug and vector release. -Cationic properties may cause cytotoxicity.
Polymeric Micelles	-Self-assembled nanoscale structures enhance the solubility and stability of hydrophobic drugs.	-Potential cytotoxicity. -Low loading efficiency.
Poly (beta-amino esters)	-Biodegradable, ensuring safe elimination from the body post-therapy. -Lower cytotoxicity compared to other vectors. -High transfection efficiency. -Facilitates controlled and sustained release of drugs or genes over time.	-Requires optimization to balance degradation rates and therapeutic delivery.

**Table 3:** Various known viral vectors and transgenes for gene therapy of GBM.

Vector/Gene Therapy Agent	Mechanism of Anti-GBM Effect
Retro- or adenovirus/HSV-tk [53].	Converts ganciclovir to the antiviral drug ganciclovir triphosphate.
Retrovirus/Toca511 [54].	Converts prodrug 5-FC to anti-neoplastic 5-FU.
Adenovirus/SCH-58500 [55].	Tumor suppressor gene therapy transfects the tumor cell with the missing p53 gene.
Adenovirus/Ad-p53 [55]	Tumor suppressor gene therapy transfects the tumor cell with the missing p53 gene.
Adenovirus/AdV-tk [56]	Gene-mediated cytotoxic therapy converts valacyclovir to antiviral drug acyclovir.
Lentivirus/based doublecortin (DCX) [57].	Direct local delivery of lentivirus-based DCX gene therapy is a potential differentiation-based therapeutic approach for GBM treatment.

**Table 4:** Known non-viral vectors and transgenes for gene therapy of GBM.

Vector	Description/Mechanism
Liposome/SGT-53.	Tumor suppressor gene therapy transfects p53 gene. Restoring p53 function by SGT-53 boosts antitumor immunity, augments anti-PD1 therapy, sensitizes tumors otherwise insensitive to anti-PD1 immunotherapy and reduces immune-related adverse events [39].
Spherical Gold and Nucleic Acid NP (NU-0129) [58].	RNA interference gene therapy transfects tumor cells with siRNA targeting oncogene Bcl2-L12
PAMAM (Polyamidoamine) Cationic Dendrimers [59,60].	Dendrimers form complexes with negatively charged nucleic acids and deliver gene therapy to glioma by penetrating cellular and endosomal membranes and crossing the BBB. They can deliver various nucleic acids, including antisense oligonucleotides, microRNAs, siRNAs, and genes (DNA) into glioma cells.
Dendrigraft poly-l-lysine (DGL) [61,62].	DGL has many external amino groups for binding nucleic acids for gene therapy. DGL is conjugated to transferrin- or laminin-targeted peptides that facilitate the DGL-conjugates’ passage through the blood–brain barrier and glioma targeting.
Polymeric Micelles [63,64].	Micelles are amphiphilic copolymers whose cores can carry gene therapy agents. Micelles of cationic and hydrophobic polymer components can deliver negatively charged nucleic acids and hydrophobic cancer drugs, respectively.
PBAEs (Poly-β-amino esters) [65].	PBAEs are cationic polymers designed for gene delivery. They are biodegradable and have low cytotoxicity. Extensive polymer libraries can be synthesized using combinatorial chemistry to make PBAE polymers with various amine compositions. High-throughput screening of hundreds of PBAE polymers can find the best vectors for gene delivery. PBAE vectors can transfect up to 90% of primary GBM cells and silence up to 85% of genes with minimal cytotoxicity.

**Table 5:** Other nonviral vectors used in gene therapy for GBM.

Nanoparticles	Gene Therapy	Mechanism of Action	Surface Modification	Particle Size (nm)	Combination Therapy	Reference
NU 0129	siRNA	Targets oncogene Bcl2-L12	See details under combination therapy section.	Exact size information not available.	NU-0129 drug product consists of 25 mg of conjugated drug substance (0.987 mg of Bcl2L12 siRNA duplex) and 120 mg of D-mannitol (USP)	[22,66]
RGD	TRAIL	Binds to integrin α(v)β(3)	PEG-PEI	10 nM	Paclitaxel	[33]
PU	miR145	Targets Oct4 and Sox2	PU-PEI	N/A	Radiation and TMZ	[34,35]
ECHO	SiRNA	Anti-HIF-1alpha	EHCO/siRNA, RGD−PEG/EHCO/siRNA, BN−PEG/EHCO/siRNA and mPEG/EHCO/siRNA	179 ± 9, 184 ± 6, 170 ± 10 and 186 ± 9	-	[34,38]
SGT53	Wild-type P53 Plasmid DNA	Restoring P53 function sensitizes refractory tumors to anti-PD1 antibodies	N/A	114.4 ± 8.4 nm	N/A	[22,39]
Albumin-SPNP	siRNA	Downregulates STAT3	RGD	115 ± 23.4	Radiation	[40]
HDL-SPNP	CpG	Toll-like receptor 9 (TLR9) agonist increases anti-tumor CD8+ T cell responses	DTX	8–12 nm	Docetaxel, Radiation	[41]
Iron oxide	siRNA	knockdowns the GFP transgene expression in C6/GFP+ cells	PEG-PEI-CTX	7.5 nm	Chlorotoxin	[44]
PAMAM	Antisense oligonucleotides, microRNAs, siRNAs, and genes	N/A	histidine and arginine; PAMAM-PEG conjugated with transferrin, chlorotoxin, or Angiopep-2	1 to 13 nm	N/A	[22]
Dendrigraft poly-l-lysine (DGL)	pORF-hTRAIL or survivin pcDNA3.1-ING4	Tumor suppressor gene inhibitor for growth 4	transferrin- or laminin-targeted peptides	30–158 nm	doxorubicin	[22]
Polymeric Micelles (folate)	TRAIL; BCL-2 siRNA; pORF-hTRAIL	N/A	FA-PEG-PEI; FA-PEI; RGD-PEG-PEI	N/A	CD/5-FC; Doxorubicin; paclitaxel	[22]
Poly(β-amino ester)	DNA	N/A	PEG	N/A	ganciclovir	[22]

N/A: Information not available.

## 5. Discussion

Viral and nonviral vector-based genetic therapies aim to improve the quality of life and life expectancy of GBM patients. GBM, particularly temozolomide-resistant GBM, has a poor prognosis, and significant treatment advances are needed. GBM has excellent targets for gene therapy that may be exploited to improve GBM outcomes [23]. Several clinical gene therapy trials for GBM have been completed, and others are being developed. Primary therapies and their associated vectors and genetic cargoes are described in Table 1, Table 2 and Table 3.

Viral and nonviral vectors are nanoparticles. Nanoparticles are prone to scavenging by the reticuloendothelial system, particularly after systemic delivery. Nonviral vectors may be coated with PEG or PEI to enhance their functionality, stability, biocompatibility, and effectiveness for targeted drug and gene delivery applications.

Recently, new innovative therapies, including CAR-T Cell Therapy, immune checkpoint inhibitors, and Tyrosine Kinase Inhibitors, have gained more traction with ongoing research. Based on a recent review article by Frumento D. et al., from 2018 to 2024, multiple combinations have undergone trials, including Axitinib, Lomustine, Bevacizumab, Buparlisib, Temozolomide, and other agents. There are ongoing trials for GBM using Entrectinib plus Ibrutinib and Indoximod [67]. Additionally, NK cell inhibitor therapy, described in a recent article by Greppi et al., is also a promising future GBM treatment, which requires further research and evaluation in clinical trials [68]. Finally, neoantigen-based therapy is an area of intense research interest. A recent article by Weng C. et al. describes ongoing clinical trials of personalized tumor-specific neoantigen-based therapy. Some of these trials are used in combination with standard GBM therapy [69].

Gene therapy acts through transgene and immune-mediated pathways to kill GBM tumor cells. Enhancing the tumor cytotoxicity of gene therapy while avoiding neurological deficits is critical in preserving the quality of life and prolonging the life expectancy of GBM patients. Viral and non-viral-vector-delivered gene therapies are being investigated in preclinical and clinical studies. The immune reaction to gene therapy vectors in GBM patients must be suppressed until the transgene can be expressed and have cytotoxic anti-tumor effects. After that, pharmacologic immune suppression can be removed to allow the viral vector and tumor cell fragments to stimulate immune reactivity in the tumor environment. Further research in gene therapy can lead to the development of new therapeutic options for patients who fail standard therapy. Genetic therapies presently employ direct injection of a viral vector into the GBM. Less invasive innovative methods are being developed to deliver gene therapy agents to tumors. Successful intravenous gene therapy for GBM requires effective transit of the gene therapy vector to the GBM through the blood–brain and blood–tumor barriers.

## 6. Conclusions

Gene therapy vectors, which deliver genetic payloads such as DNA transgenes and RNA inhibitors, offer significant potential for targeted anti-GBM therapies. Viral oncolytic therapies also produce unique cytolytic and immunogenic anti-GBM effects. The cytotoxic and immunogenic effects of genetic and viral agents hold promise for improving treatment efficacy, particularly in overcoming the challenges of GBM’s aggressive nature and resistance to conventional therapies. As research in gene therapy and virotherapy for GBM continues to advance, these approaches could play a pivotal role in developing more effective, personalized treatment options.

## Data Availability

Not applicable.
